# Contrasting Life Histories in Neighbouring Populations of a Large Mammal

**DOI:** 10.1371/journal.pone.0028002

**Published:** 2011-11-18

**Authors:** Tom H. E. Mason, Roberta Chirichella, Shane A. Richards, Philip A. Stephens, Stephen G. Willis, Marco Apollonio

**Affiliations:** 1 School of Biological and Biomedical Sciences, University of Durham, Durham, County Durham, United Kingdom; 2 Adamello Brenta Nature Park, Strembo, Trento, Italy; 3 Department of Zoology and Evolutionary Genetics, University of Sassari, Sassari, Sardinia, Italy; Ohio State University, United States of America

## Abstract

**Background:**

A fundamental life history question is how individuals should allocate resources to reproduction optimally over time (reproductive allocation). The reproductive restraint hypothesis predicts that reproductive effort (RE; the allocation of resources to current reproduction) should peak at prime-age, whilst the terminal investment hypothesis predicts that individuals should continue to invest more resources in reproduction throughout life, owing to an ever-decreasing residual reproductive value. There is evidence supporting both hypotheses in the scientific literature.

**Methodology/Principal Findings:**

We used an uncommonly large, 38 year dataset on Alpine chamois (*Rupicapra rupicapra*) shot at various times during the rutting period to test these two hypotheses. We assumed that body mass loss in rutting males was strongly related to RE and, using a process-based approach, modelled how male relative mass loss rates varied with age. For different regions of our study area, we provide evidence consistent with different hypotheses for reproductive allocation. In sites where RE declined in older age, this appears to be strongly linked to declining body condition in old males. In this species, terminal investment may only occur in areas with lower rates of body mass senescence.

**Conclusions/Significance:**

Our results show that patterns of reproductive allocation may be more plastic than previously thought. It appears that there is a continuum from downturns in RE at old age to terminal investment that can be manifest, even across adjacent populations. Our work identifies uncertainty in the relationship between reproductive restraint and a lack of competitive ability in older life (driven by body mass senescence); both could explain a decline in RE in old age and may be hard to disentangle in empirical data. We discuss a number of environmental and anthropogenic factors which could influence reproductive life histories, underlining that life history patterns should not be generalised across different populations.

## Introduction

In most stable populations, reproductive life histories should be optimised to maximise lifetime reproductive success [1], [2]. Accordingly, the question of how long-lived iteroparous animals should allocate resources to reproduction over their lives is of great ecological importance [1]–[3]. Trade-offs exist not only between survival and reproduction, but also between current and future reproduction [1]–[5]. The allocation of resources to current reproduction is likely to be damaging to future reproductive potential [3]. As such, a breeding individual must carefully balance yearly investment in reproduction against future reproductive potential in order to maximise lifetime reproductive success.

The classical view of reproductive allocation in iteroparous species is that reproductive effort (RE; the allocation of resources to current reproduction rather than to future reproduction, growth or survival) should increase throughout life following maturity, due to a decreasing residual reproductive value [2]–[5]. This has been termed the terminal investment hypothesis [4]. The hypothesis predicts that the trade-off between current and future reproduction becomes less relevant in later life as the potential for future reproduction diminishes. As such, RE is predicted to peak in old individuals in their final breeding seasons. There is support for terminal investment from a range of taxa [6]–[9] although, to date, there is little evidence for male terminal investment in natural populations [e.g. 10].

Evidence from a number of species suggests that RE peaks at prime-age (here defined as the age of peak body condition) and subsequently decreases [11]–[14]. This could either arise as a by-product of reproductive senescence [15] or could be a life history tactic designed to maximise RE over a number of years. Specifically, a large allocation of resources to current reproduction might be sub-optimal if it is likely to cause death. Instead, individuals might show reproductive restraint, eking out future reproduction over a number of years [14], [16]. The results of a recent modelling study by McNamara et al. [16] suggest that reproductive restraint and terminal investment should be favoured in different conditions, depending on the rates of intrinsic and extrinsic mortality (due, for example, to disease or predation). This raises the intriguing possibility that, if rates of intrinsic and extrinsic mortality vary among populations, different patterns of reproductive allocation could exist within a species.

Ungulate species provide good model systems with which to study the allocation of RE over time. Males and females generally have strongly contrasting life histories, investing in reproduction in different ways and over different periods of the reproductive cycle [1], [17]. Females invest heavily in gestation, lactation and parental care, with their reproductive success strongly dependent on raising young successfully [18]. In contrast, male reproductive success usually depends on competing for access to females during a short rutting season [19]. Specifically, male reproductive behaviour consists of agonistic interactions with rival males and courtship behaviours to attract females [14], [20], [21]. Male reproductive allocation remains relatively understudied, but can be investigated by measuring the somatic costs of reproductive behaviour during the rut. Rutting male ungulates often adopt a strategy of ‘appetite suppression’, which means that they lie towards the capital end of the continuum between capital and income breeding, relying on stored energy to breed [22]–[24]. As such, the proportion of body mass lost by a male during the rut can be used as an estimate of RE [25]. Relative mass loss is an informative indicator of RE because there is known to be an important trade-off between body mass and subsequent over-winter survival for mammals living in unpredictable environments [26], [27].

Reproductive allocation by males has been studied in several ungulates, using both transversal (cross-cohort) and longitudinal approaches. Using a transversal hunting dataset, Yoccoz et al. [14] found that RE peaked in prime-aged male red deer (*Cervus elaphus*) before declining; this is probably explained by a reduced ability of older males to hold a harem [14], [17]. Declines in effort in older individuals have also been observed in longitudinal studies on male bison (*Bison bison*) [28] and mountain goats (*Oreamnos americanus*) [13]. Intriguingly, Mysterud et al. [29] used a transversal analysis to put forward evidence ‘consistent with the terminal investment hypothesis’ of increasing RE with age in adult male moose (*Alces alces*), although the authors noted that effort tended to stabilise or decrease in the oldest individuals (which were sparse in their dataset). Unfortunately, several of these studies were based on small sample sizes, particularly of individuals of prime-age and older, a common problem in life history studies [30]. A consequence is that earlier studies have fitted RE to data from age classes with only one or two individuals, inevitably constraining the inferences that can be made from the data [14], [29].

Here, we analyse the relationship between male age and RE using a large transversal dataset of Alpine chamois (*Rupicapra rupicapra*). During the rut, males allocate most of their non-rutting time to rest rather than foraging; they are thus capital breeders [24]. We tested for variation in male RE with age using the mean, relative rate of body mass loss during the rut, as an estimate of RE [14], [29]. Rather than taking repeated measurements of the same individuals, we estimated mass loss across different individuals shot at different times during the season. Also, we predicted age-related patterns of body mass both before and after the rut, as these could reveal important life history characteristics which influence the allocation of RE. Using a process-based model of mass loss, we assessed how RE varied with age among our sites and between years. In contrast to previous studies [e.g. 29], we fitted RE to data directly, rather than indirectly inferring RE from body mass predictions at different times; this improved our ability to estimate uncertainty in the RE estimates.

## Materials and Methods

### Ethics Statement

Our data collection complied with all relevant national, regional and provincial Italian laws.

### Data Collection

Data were collected in the Central-Eastern Italian Alps, across a 1,333 km^2^ area of Trento Province (46°02′N, 10°38′E). The elevation of the study area ranges from 52 to 3,558 m above sea level, with a mean altitude of 1,586 m. It is forested up to the tree-line at about 2,000 m, above which habitat consists of Alpine meadows and open rock faces. The study area consists of six chamois hunting districts (Fig. S1) which are subdivided further into 68 municipal reserves. Mountain-dwelling ungulates, chamois are polygynous [31], [32] and yet, in terms of horn and body size [33]–[35], relatively sexually monomorphic (although body mass dimorphism does increase considerably before the rut [36]). Breeding male chamois defend small clustered territories during the rutting season, excluding rival males and defending oestrus females [31], [35]. Male rutting behaviour consists of agonistic interactions with rivals such as chasing and posturing; and interactions with sexually mature females, including herding and copulation [35]. Chamois are hunted with rifles every year from mid-September to the end of December. Hunting is controlled through licenses issued by local wildlife boards. Area-wide hunting quotas are set for specific age classes in each sex (Table S1). There is little potential for artificial selection by hunters (e.g. by shooting the largest individuals at the start of the season) because flight distances (distance from hunter when chamois takes flight) in these hunted populations are large and, due to the generally open habitat, hunters can be easily detected. As such, whilst trophy hunting is practised in Trento Province, hunters have limited shooting opportunities per day and will typically shoot the first animal of a suitable age class that they encounter. Also, since both males and females are trophy hunted, we would expect to see pronounced patterns of decreasing mass with season in adults of both sexes if artificial selection was occurring. This is not the case in females. Furthermore, we found no evidence of hunter selection for larger bodied age classes earlier in the season, which might be the case if there was strong hunter selection for larger body mass (Fig. S2).

Data were collected on the eviscerated body mass, sex, age and date shot of 28,966 Alpine chamois (15,155 males and 13,811 females) culled over 38 consecutive hunting seasons between 1973 and 2010. Ages were estimated from counts of horn growth annuli [37]. Males varied between 1 and 19 years old and females varied between 1 and 21 years old. Dates of shooting were converted to Julian day of year and ranged from day 247 to 365. We analysed data from three of the six hunting districts in our study. The remaining three districts contained too few data to assess RE reliably across all ages. Also, data collected prior to 1979 were limited, so these years were excluded from our analysis. Although timing of the rut is likely to vary slightly between sites and years, we had insufficient data to assess this reliably. Therefore, based on extensive field observations, we assumed a fixed rut period of days 300 to 340. Given the strict criteria set out above, our sample size was 7,202 males and 6,415 females (see Table S2).

### Statistical Analysis

We sought evidence for age-dependence in the relative rate of male mass loss during the rutting period. We recognise that annual population density is a potentially important factor affecting the rate of mass loss and may vary across years at a given site [14]. For this, we used as a proxy the number of individuals culled each year divided by the area of suitable habitat in each site. This proxy, *d*, is only a crude index but generally correlates well with population density values estimated from censuses performed since 1992 (Fig. S3). We developed a modelling framework that allows age-dependent change in mass either to vary annually in response to changes in *d*, or to exhibit a smooth trend across years. We assumed that, during the rutting period of year *y*, individuals of age *a* lose mass at mean, relative rate 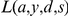, when in a population at density *d* and in a given site *s*. As *L* is independent of body mass, it can be used to compare RE directly across sexes and age-classes. Specifically, if the expected mean mass of individuals on day 300 (hereafter, the ‘initial mass’) is 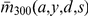, then their expected mass on day *t* (*t*≥300) is
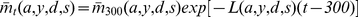
(1)


Our objective is to estimate 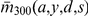 and 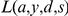 using model selection to assess the importance of age, year, population density, and site. Using these estimates we could infer the expected mean mass of individuals at the end of the rut, on day 340 (hereafter, ‘final mass’).

We assumed that age-dependence for both the initial mass and mass loss functions could be characterised using cubic splines. Cubic splines were used to avoid numerical instabilities that are often characteristic of higher order polynomials [e.g. 38]. We assumed that the shape of these splines was fixed across years for any site (i.e. age-dependence was fixed) but that their relative magnitude could vary among years to reflect annual environmental variation. Specifically, we used three cubic splines, each spanning 4 years of age, to describe complete age-dependency from 1 to 13 years of age. These three splines correspond broadly to pre-prime age (1–4 years), prime-age (5–9 years) and post-prime age (10–13 years). There were too few individuals older than 13 within each site to permit reliable predictions for these individuals. Using fewer than three splines resulted in a poor fit to our data, whereas using more than three splines did not improve the fit for either function. Initial mass, and mass loss rate, were described by
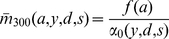
(2a)


and



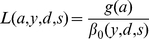
(2b)where *f*(*a*) and *g*(*a*) describe the two three-piece, cubic-spline functions and satisfy *f*(1) = *g*(1)  = 1. Thus, the scaling parameter *α_0_* represents the inverse of the initial mass of the youngest age modelled (yearlings). Similarly, *β_0_* describes the inverse of the rate of mass loss of yearlings. To identify long-term trends and density-dependence, models were constructed that allowed *α_0_* and *β_0_* to be either: constant, linearly dependent on *d*, linearly dependent on *y*, or quadratic in *y*. Incorporating yearly trends and density-dependence in life history traits is plausible for long-term studies of mammalian life histories. In total, we fitted 16 models to both male and female mass time-series data for each of the three sites (Tables 1 & 2).

**Table 1:** Model selection results from cubic spline model fitted to male chamois body mass data.

Model	K	Adamello		Presanella		Brenta	
		LL	ΔAIC	LL	ΔAIC	LL	ΔAIC
*N*		3539		1426		2237	
M(*α*_0_, *β*_0_)	13	−8625.5	82.6	−3629.6	106.2	−5501.1	53.0
M(*α*_0_, *β*_0_(y))	14	−8621.8	77.3	−3602.6	54.2	−5487.4	27.6
M(*α*_0_, *β*_0_(y^2^))	15	−8621.8	79.3	−3584.6	20.1	−5483.9	22.5
M(*α*_0_, *β*_0_(d))	14	−8624.7	83.0	−3602.8	54.4	−5493.9	40.6
M(*α*_0_(y), *β*_0_)	14	−8597.2	28.1	−3581.3	11.5	**−5474.5**	**1.8**
M(*α*_0_(y), *β*_0_(y))	15	**−8582.6**	**0.9**	−3581.3	13.6	−5474.5	3.8
M(*α*_0_(y), *β*_0_(y^2^))	16	**−8581.2**	**0.0**	−3576.9	6.7	−5474.1	5.0
M(*α*_0_(y), *β*_0_(d))	15	−8594.6	24.8	**−3574.6**	**0.0**	−5474.5	3.7
M(*α*_0_(y^2^), *β*_0_)	15	−8597.2	30.1	−3578.3	7.5	**−5472.6**	**0.0**
M(*α*_0_(y^2^), *β*_0_(y))	16	−8582.5	2.7	−3578.3	9.5	−5471.9	0.6
M(*α*_0_(y^2^), *β*_0_(y^2^))	17	−8580.7	1.1	−3578.2	11.4	−5471.6	2.0
M(*α*_0_(y^2^), *β*_0_(d))	16	−8593.8	25.2	−3573.9	0.7	−5471.6	0.0
M(*α*_0_(d), *β*_0_)	14	−8606.9	47.4	−3586.8	22.6	−5483.3	19.4
M(*α*_0_(d), *β*_0_(y))	15	−8606.9	49.4	−3578.0	6.9	−5479.0	12.8
M(*α*_0_(d), *β*_0_(y^2^))	16	−8601.4	40.4	−3576.7	6.2	−5479.0	14.8
M(*α*_0_(d), *β*_0_(d))	15	−8590.4	16.4	−3585.8	22.5	−5483.0	20.7

Models are distinguished by the functional forms of *α*_0_ and *β*_0_. Specifically, we allowed *α*_0_ and *β*_0_ to be constant across years (*α*_0_; *β*_0_), vary linearly with year (*α*_0_(y); *β*_0_(y)), quadratically with year (*α*_0_(y^2^); *β*_0_(y^2^)) or linearly with population density (*α*_0_(d); *β*_0_(d)). Maximum log-likelihoods (LL) and ΔAICs are shown for each site. The most parsimonious models for each site are highlighted in bold (i.e. have a ΔAIC value that is ≤6 and lower than all simpler nested versions; see Richards [40]). *n* is sample size for each site.

**Table 2:** Model selection results from cubic spline model fitted to female chamois body mass data.

Model	K	Adamello		Presanella		Brenta	
		LL	ΔAIC	LL	ΔAIC	LL	ΔAIC
*N*		2990		1329		2096	
M(*α*_0_, *β*_0_)	13	−7092.9	78.4	−3187.6	95.7	−5002.0	25.2
M(*α*_0_, *β*_0_(y))	14	−7076.4	47.4	−3155.7	34.0	−4997.1	17.5
M(*α*_0_, *β*_0_(y^2^))	15	−7062.0	20.6	−3147.8	20.1	−5000.5	26.2
M(*α*_0_, *β*_0_(d))	14	−7083.5	61.5	−3174.9	72.3	−5002.5	28.4
M(*α*_0_(y), *β*_0_)	14	−7073.3	41.1	−3142.8	8.1	−4995.1	13.5
M(*α*_0_(y), *β*_0_(y))	15	−7063.6	23.8	**−3138.5**	**1.7**	−4995.3	16.0
M(*α*_0_(y), *β*_0_(y^2^))	16	−7063.5	25.6	**−3138.4**	**3.5**	−4992.1	11.4
M(*α*_0_(y), *β*_0_(d))	15	−7066.2	29.0	−3140.8	6.3	−4995.1	15.4
M(*α*_0_(y^2^), *β*_0_)	15	−7073.6	43.8	−3142.7	9.9	**−4989.0**	**3.2**
M(*α*_0_(y^2^), *β*_0_(y))	16	−7061.2	21.0	**−3136.7**	**0.0**	−4988.9	5.1
M(*α*_0_(y^2^), *β*_0_(y^2^))	17	−7060.5	21.6	−3136.4	1.4	−4989.5	8.3
M(*α*_0_(y^2^), *β*_0_(d))	16	−7071.3	41.3	−3139.9	6.4	**−4986.4**	**0.0**
M(*α*_0_(d), *β*_0_)	14	−7068.0	30.7	−3168.8	60.2	−4999.4	22.1
M(*α*_0_(d), *β*_0_(y))	15	−7066.7	30.1	−3151.2	27.0	−4998.1	21.4
M(*α*_0_(d), *β*_0_(y^2^))	16	**−7050.7**	**0.0**	−3147.5	21.7	−4997.6	22.4
M(*α*_0_(d), *β*_0_(d))	15	−7060.5	17.5	−3167.4	59.4	−4998.4	22.0

Models are distinguished by the functional forms of *α*_0_ and *β*_0_. Specifically, we allowed *α*_0_ and *β*_0_ to be constant across years (*α*_0_; *β*_0_), vary linearly with year (*α*_0_(y); *β*_0_(y)), quadratically with year (*α*_0_(y^2^); *β*_0_(y^2^)) or linearly with population density (*α*_0_(d); *β*_0_(d)). Maximum log-likelihoods (LL) and ΔAICs are shown for each site. The most parsimonious models for each site are highlighted in bold (i.e. have a ΔAIC value that is ≤6 and lower than all simpler nested versions; see Richards [40]). *n* is sample size for each site.

When fitting the models we assumed that variation about the predicted mean (eqn. 1) was normally-distributed. Models most consistent with the data were selected using Akaike's Information Criterion (AIC; [39]). To avoid selecting overly complex models we used the two-step selection criterion suggested by Richards [40] . First, all models having an AIC within six units of the smallest AIC calculated were selected (i.e. ΔAIC≤6). Second, in order to remove overly complex models, we disregarded those that had a higher AIC value than any simpler nested model.

**Figure 1:**
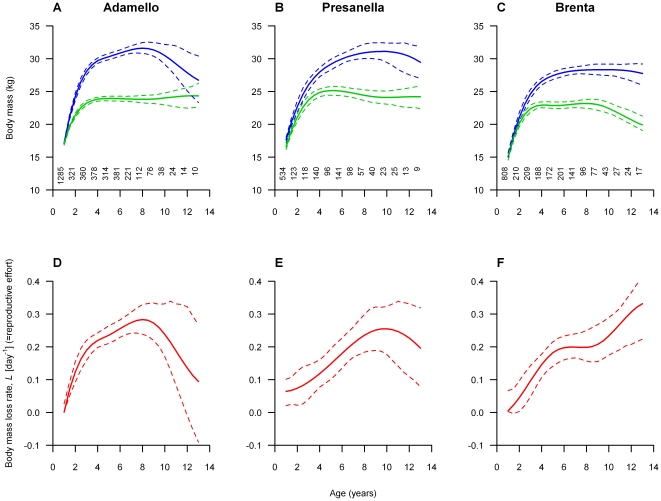
Patterns of male mass and mass loss with age. Variation in mean predicted male chamois initial body mass (blue lines), final body mass (green lines) and body mass loss rate, *L* (red lines), with age across our three study sites; Adamello (A & D), Brenta (B & E) and Presanella (C & F). Predicted values for each age are mean values across all years. Dashed lines represent 95% confidence intervals from 1,000 bootstrapped replicates [41]. Sample sizes are displayed for each age.

**Figure 2:**
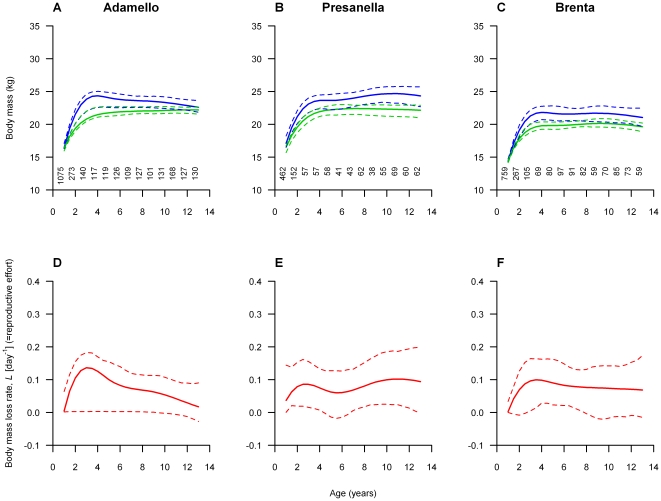
Patterns of female mass and mass loss with age. Variation in mean predicted age of female chamois initial body mass (blue lines), final body mass (green lines) and body mass loss rate, *L* (red lines), across our three study sites; Adamello (A & D), Brenta (B & E) and Presanella (C & F). Predicted values for each age are mean values across all years. Dashed lines represent 95% confidence intervals from 1,000 bootstrapped replicates [41]. Sample sizes are displayed for each age.

Ignoring site effects substantially reduced the fit of our models, justifying fitting separate models for each site (Tables S3 & S4). For each site, we fitted the most parsimonious model to 1,000 bootstrapped replicates [41], stratified by age, to determine 95% confidence intervals for initial mass, final mass and rate of mass loss (see Figs. 1 & 2). Statistical analyses were performed using R version 2.12.1 [42]. Results are reported with standard errors, where applicable.

## Results

For males, our best AIC models explained a large proportion of the variation in our data (Fig. S4). For each site, the scaling parameter associated with initial mass, *α_0_,* varied with year (Table 1), indicating long-term population change. The best fitting function varied between sites (quadratic in Adamello and Brenta but linear in Presanella) but the overall trends are surprisingly consistent: males have become consistently lighter in all sites across the study period (Fig. S5); for example, yearling males have become between 1.1 and 2.4 kg lighter over the past 32 years. These changes may be a result of higher competition, as our data suggest that population density has increased over this period.

Model selection indicated that *β*_0_ was influenced by site and year and *g*(*a*) differed between sites (Table 1), which suggests that the patterns of net rate of relative mass loss, *L*, differed across sites and have changed during the 30 years of the study. However, despite such differences there was clear evidence of a somatic cost of reproduction for males during the rut across all ages and sites (mean adult male proportional mass loss: Adamello, 19.1±1%; Presanella, 17.1±1.1%; Brenta, 18.1±1.3%; Fig. 1). In contrast, female mass loss during this period was much lower (mean adult female proportional mass loss: Adamello, 7.2±0.7%; Presanella, 8.0±0.3%; Brenta, 7.7±0.2%; Fig. 2).

In adult females, initial and final mass are relatively constant with age (Fig. 2A–C). However, male mass is highly age-dependent and the shape of this age-dependency varies among sites. Interestingly, initial mass shows a strong decline after 8 years in Adamello (Fig. 1A) and a weaker decline after 10 years in Presanella (Fig. 1B). However in Brenta, initial mass remains relatively constant throughout adulthood (Fig. 1C). The age-related pattern of final mass also varies among sites. In Adamello and Presanella, adult males show a constant final mass (Fig. 1A & B); however, in Brenta, final mass declines after 9 years (Fig. 1C).

In females, confidence intervals suggest that there are no strong patterns of relative mass loss with age (Fig. 2). In contrast, male relative mass loss is highly dependent on age and there is considerable variation in the shape of this relationship among sites (Fig. 1). In Adamello, rate of mass loss initially increases rapidly with age, peaking in 8 year olds before dropping (Fig. 1D). In Presanella, rate of mass loss increases more gradually, peaking at 10 years before apparently dropping (Fig. 1E). In Brenta, after a slight plateau between 5 and 9 years, rate of mass loss continues to increase steadily through older ages (Fig. 1F). In all sites, confidence intervals widen with increasing age (due to decreasing sample size); however, the general trends of mass loss with age in Adamello and Brenta appear robust (Fig. 1D & F). In Presanella, whilst our best model predicts a decrease in mass loss after 9 years, due to the width of confidence intervals at old age we cannot rule out the possibility that RE levels off (Fig. 1E).

**Figure 3:**
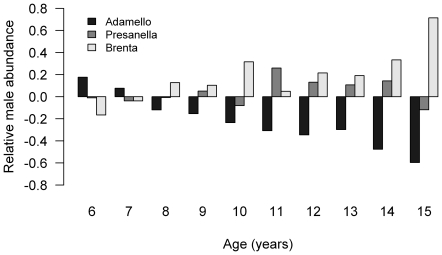
Age structure of males. The abundance of males in each site at each age, from 6 years upwards (hunting age class I, see Table S1), relative to the area-wide age-distribution. Assuming that individuals within each hunting age class are shot randomly, the proportions of different ages shot represent the age structure of that class. A value of 0 indicates no difference to the mean population age-structure. Adamello is a young population relative to Brenta and Presanella.

Variation in male age structure among the sites shows that there are increasingly fewer males in Adamello from 8 years onwards, relative to Presanella and Brenta (Fig. 3). This suggests that the survival of older males is lower in Adamello than in the other sites. 6 y.o. males in Adamello live an average of 1.3 additional years compared to 1.7 and 2 more years in Presanella and Brenta, respectively.

## Discussion

Male chamois suffer a substantial somatic cost during the rut, with breeding males losing as much as 28% of their body mass (Fig. 1). Unsurprisingly, female relative mass loss during this same period is considerably lower and less affected by age (Fig. 2), underlining that male mass loss is a consequence of RE. How males allocate resources to reproduction across their lives varies considerably among sites (Fig. 1D–F). Within two of our sites there are declines in RE after prime-age, whereas males in the other site appear to show terminal investment. Several aspects of male life history, including rates of decline in initial mass at old age and the consistency of final mass with age, differ substantially across the study area, strongly influencing the patterns of RE. Such variation between neighbouring populations is surprising.

In Adamello and Presanella, RE peaks at prime-age before decreasing in old individuals (Fig. 1D & E). The downturn in RE at old age is particularly pronounced in Adamello. Studies of other male ungulates have found similar results [13], [14], [28], as have studies on males from other taxa [11]. This pattern is consistent with the predictions of reproductive restraint but could also arise simply as a by-product of body mass senescence. That the initial mass of males strongly decreases after prime-age, particularly in Adamello (Fig. 1A & B), indicates such senescence (decline in fitness due to physiological degradation [43]). This is likely to hinder the ability of old males to defend oestrus females as large body size is important for male:male competition in polygynous ungulates [e.g. 20,44]. As such, we might expect that old chamois adopt alternative mating strategies, as seen in red deer [17]. The pattern of final mass with age also influences the pattern of male reproductive allocation in these sites. Final mass is largely unaffected by age among adult males in Adamello and Presanella (Fig. 1A & B), hinting at a ‘giving-up mass’ which could be important for over-winter survival [26], [27]. This would further constrain the ability of males to invest resources in reproduction, consistent with the reproductive restraint hypothesis. It is, however, difficult to disentangle senescence-driven declines in RE and reproductive restraint. After all, high senescence rates in later life could be the consequence of a life history designed to maximise RE early in life. Whether an inability to compete in later life (owing to body mass senescence) is consistent with reproductive restraint is currently unclear.

In Brenta, RE appears to increase throughout life, a result consistent with terminal investment (Fig. 1F). There is evidence for terminal investment from a range of taxa [6]–[8] but our study provides some of the first evidence in males (see also [45]). The ability of old male chamois to continue to increase their RE can be partly explained by their ability to hold on to breeding territories. Data from other chamois populations suggests that males show high levels of site-fidelity year by year and profit from a ‘prior residence advantage’ in territory ownership [31]. As such, experienced males that have defended a given territory in the previous year's rut are more likely to defend it successfully in the present rut, provided they are in good condition [31]. This means that male chamois at least have the potential to increase RE throughout their lives, in contrast to species such as red deer where males past their prime cannot hold harems [17]. Unlike in the other sites, initial mass declines little after prime-age in Brenta (Fig. 1C). This means that old males in Brenta are better at acquiring resources in between rutting seasons and are likely be more competitive as a result. Perhaps unsurprisingly, terminal investment may only be optimal where old individuals are able to compete effectively with their younger rivals. Also, in contrast to the other sites, there is no consistent final mass across adult males in Brenta (Fig. 1C); old males end the rut in worse condition than young males do, presumably putting themselves at a greater risk of over-winter mortality. In line with terminal investment, the benefits of high RE would outweigh such a survival cost [2]–[5].

We have shown that several key life history characteristics vary between sites, and are important in influencing how RE is allocated with age. The variation among the age-related patterns of initial mass among our sites appears particularly important. The low initial masses of older males in Adamello and Presanella might limit their ability to invest highly in RE, even if classical life history theory suggests that they should. This, twinned with an apparent cut-off mass, appears to drive a downturn in RE at old age in these sites. Survival also appears to vary among sites. Adult male survival is considerably lower in Adamello (Fig. 3); 6 y.o. males live an average of 1.3 additional years compared to 1.7 and 2 more years in Presanella and Brenta, respectively. This, and the pronounced body mass senescence, could be linked to the apparent faster pace of life there (Fig. 1D–F). RE increases much more rapidly with age in Adamello, suggesting that males start becoming involved in the rut at a younger age (mean 3 y.o. proportional mass loss: Adamello, 17.3%; Presanella, 8.9%; Brenta, 9.2%). There may be positive feedback between shorter lifespans and a faster pace of life, which in turn results in higher rates of body mass senescence in Adamello. Senescence rates are thought to be higher in faster living species, due to a faster accumulation of damage [43]; the same might be true for faster living populations within a given species. In Brenta, where the pace of life is slower and lifespans are longer, body mass senescence is limited. It appears that only in such conditions can old males make use of their experience and continue to increase RE throughout their lives. The lower survival and faster pace of life in Adamello could be related to the higher hunting pressure there (mean proportion of adult males harvested per year (from census years): Adamello, 32.3±1.5%; Presanella, 29.5±2%; Brenta, 25.2±1.6%). Hunting has been known to influence a range of life history traits, including survival, mating behaviour and body mass [46], [47]. Where hunting pressure is higher, and risk of mortality is greater, it could pay to allocate more energy to reproduction earlier in life.

The observed variation among sites could also be mediated by environmental differences. Life histories, and specifically reproductive allocation, can be plastic with respect to the environment [48]. Brenta is a calcareous area, whilst the other sites are siliceous, harbouring very different plant communities [49]. In calcareous areas, plants are generally more nitrogen-rich [49], which could further explain the lower rates of body mass senescence in Brenta. It may be easier for older males there to acquire resources in between rutting seasons than in the siliceous sites, putting them in good stead for the rut. Differences in the patterns of final mass with age could be related to altitudinal variation among sites (mean medium elevation of shot males: Adamello, 1,979 m; Brenta, 1,675 m; Presanella, 2,182 m). Due to the considerably higher altitudes of Adamello and Presanella, their winter climates are likely to be harsher and there may be a greater risk of over-winter mortality. As such, a cut-off mass to buffer against over-winter mortality would be more important in these sites than in Brenta, thus limiting their ability to expend energy during the rut. A harsher winter climate could also explain why pre-rut breeding males weigh on average 2.7±0.16 kg and 2.12±0.07 kg more in these sites than in Brenta (Figs. 1A–C).

Our study shows that comprehensive transversal datasets can still be of great value to ecology. Whilst longitudinal field studies have the obvious advantage of being focussed on within-individual processes [30], such data are difficult to collect, frequently unavailable and, typically, limited by a focus on relatively small numbers of known individuals [e.g. 12,24]. In comparison, transversal datasets can contain information on tens of thousands of individuals; although they cannot account for individual heterogeneity, they can outline broad life history patterns at the population level. Previous studies have tended either to accept or to reject evidence of terminal investment (e.g. [14]); here, by contrast, we demonstrate for the first time that patterns of reproductive allocation can vary readily across different, and even adjacent, populations.

## Supporting Information

Figure S1 **Map of study area.** Darker shading indicates higher elevation.
(TIF)

Figure S2 **Variation in relative day shot with age in males and females.** Relative day shot was calculated for each individual by shooting day minus median day of shot animals in the same year and site. Thick lines represent median values, boxes display inter-quartile ranges (IQRs) and the extents of the vertical dashed lines show maximum and minimum values. Outliers represent values more than 1.5 of the IQR higher or lower than the mean and, in such cases, the extents of dashed lines represent maximum and minimum values within 1.5 of the IQR. Non-overlapping notches on boxes provides strong evidence that the medians of these age classes differ. Note that, if artificial selection (for larger-bodied animals) was occurring, we would expect to see a U-shaped curve in these plots (with prime-aged individuals targeted earlier in the season, and older and younger individuals typically shot later in the season); this is not the case.
(TIF)

Figure S3 **The relationship between** ***d*** **, our population density estimate, and population density estimated from censuses.** Linear regressions plotted. Fitted line equations and R^2^ values shown.
(TIF)

Figure S4 **Plots of model fit for males.** The relationship between observed body mass and predicted body mass by best model, in each site. Linear regressions plotted (red lines). Fitted line equations and R^2^ values shown. Dotted lines show 1∶1 relationship.
(TIF)

Figure S5 **Change in predicted yearling male mass over time.** Variation between predicted pre-rut body mass of yearling males (on day 300) with year in Presanella (red line), Adamello (black line) and Brenta (blue line). Line equations shown. There is a clear trend of decreasing body mass over the study period.
(TIF)

Table S1 **Sex and age-specific chamois hunting quotas in Trento province.**
(DOC)

Table S2 **Male and females data summary.**
(DOC)

Table S3 **Model selection results from cubic spline model fitted to male chamois body mass data.**
(DOC)

Table S4 **Model selection results from cubic spline model fitted to female chamois body mass data.**
(DOC)
