# Supplementary figures and images for: Contrasting Life Histories in Neighbouring Populations of a Large Mammal
Source: PLoS One. 2011 Nov 18;6(11):e28002. doi: 10.1371/journal.pone.0028002 (PMC3220718; doi:10.1371/journal.pone.0028002)

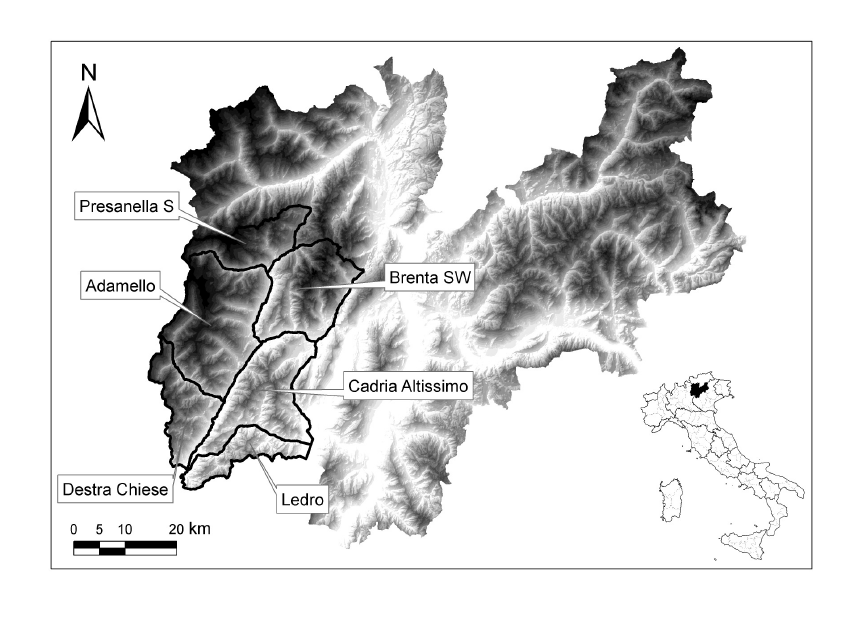

Supplement: Figure S1 — Map of study area. Darker shading indicates higher elevation. (TIF) [file pone.0028002.s001.tif]

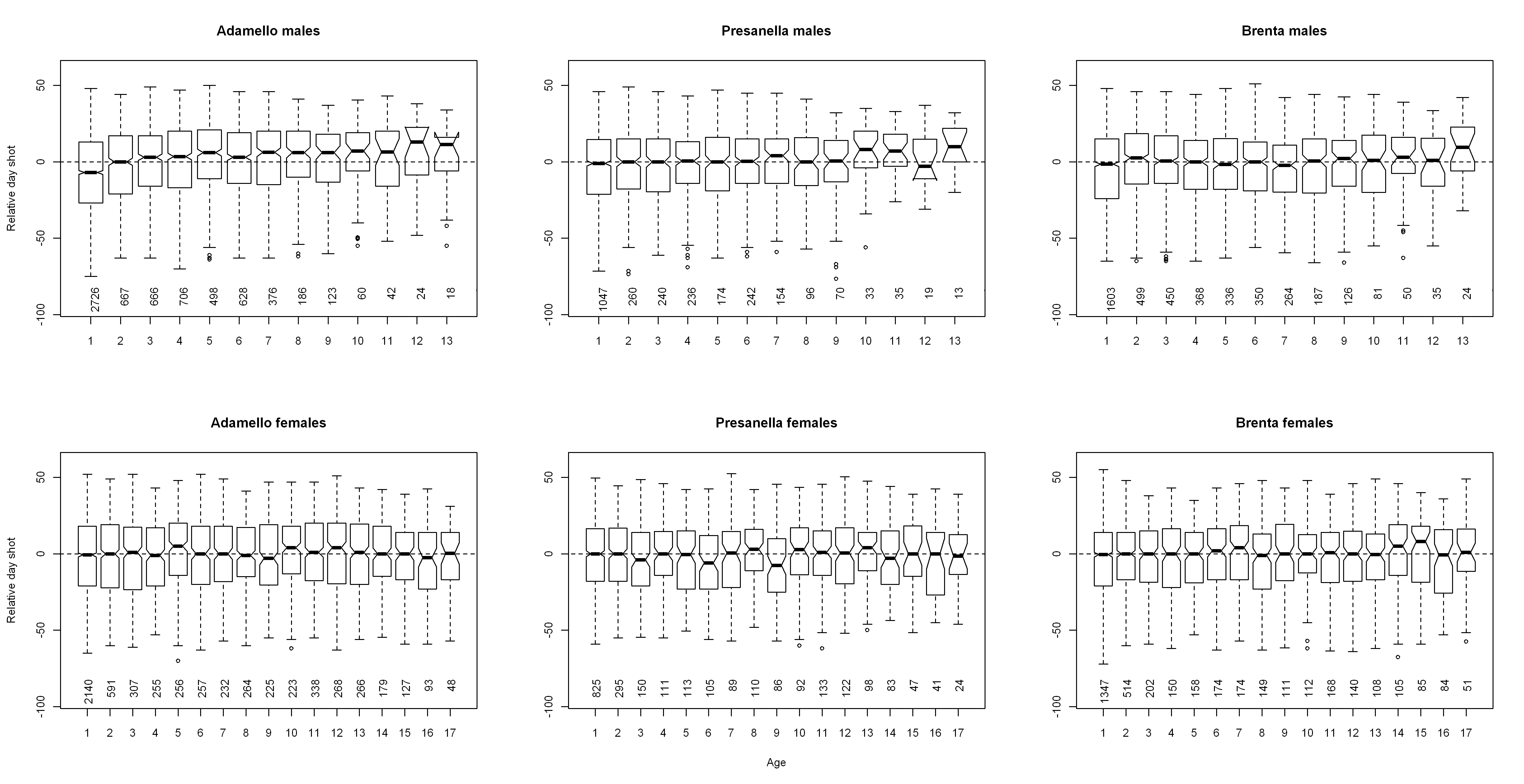

Supplement: Figure S2 — Variation in relative day shot with age in males and females. Relative day shot was calculated for each individual by shooting day minus median day of shot animals in the same year and site. Thick lines represent median values, boxes display inter-quartile ranges (IQRs) and the extents of the vertical dashed lines show maximum and minimum values. Outliers represent values more than 1.5 of the IQR higher or lower than the mean and, in such cases, the extents of dashed lines represent maximum and minimum values within 1.5 of the IQR. Non-overlapping notches on boxes provides strong evidence that the medians of these age classes differ. Note that, if artificial selection (for larger-bodied animals) was occurring, we would expect to see a U-shaped curve in these plots (with prime-aged individuals targeted earlier in the season, and older and younger individuals typically shot later in the season); this is not the case. (TIF) [file pone.0028002.s002.tif]

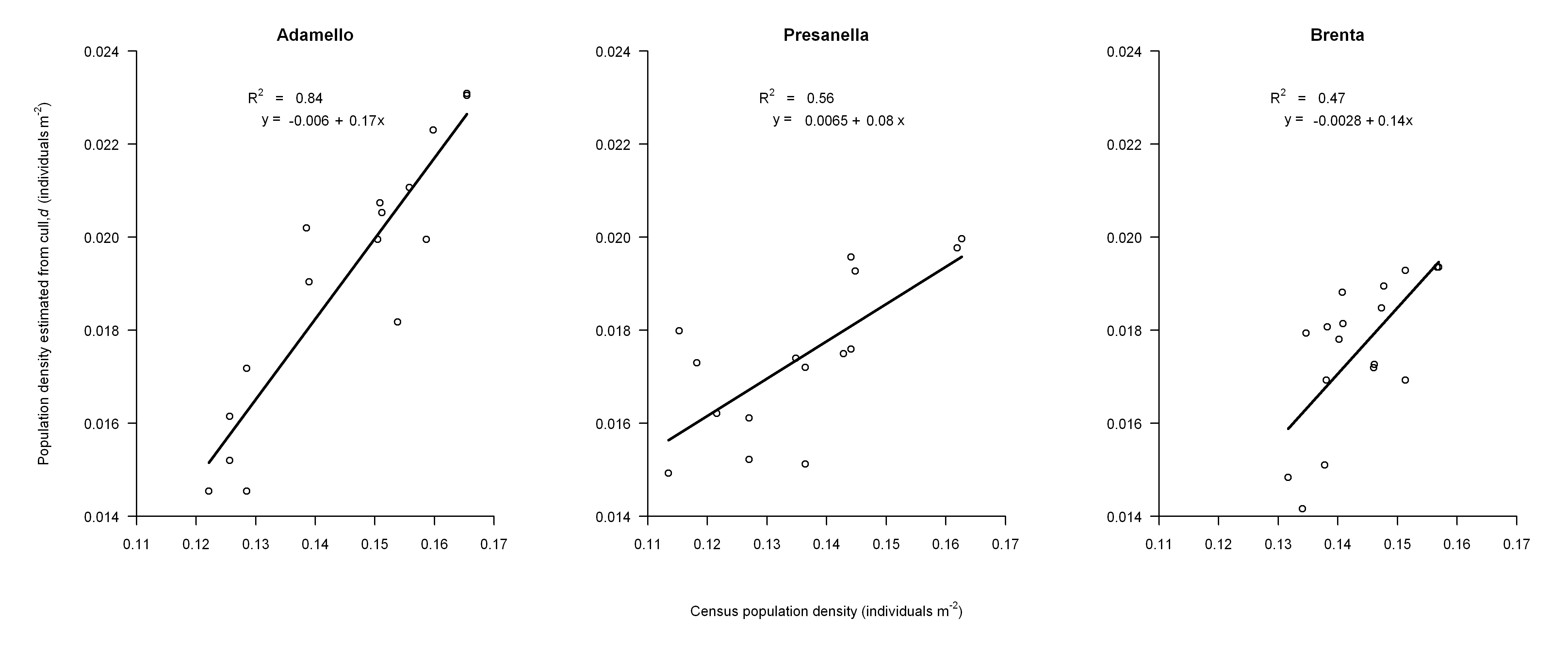

Supplement: Figure S3 — The relationship between d , our population density estimate, and population density estimated from censuses. Linear regressions plotted. Fitted line equations and R2 values shown. (TIF) [file pone.0028002.s003.tif]

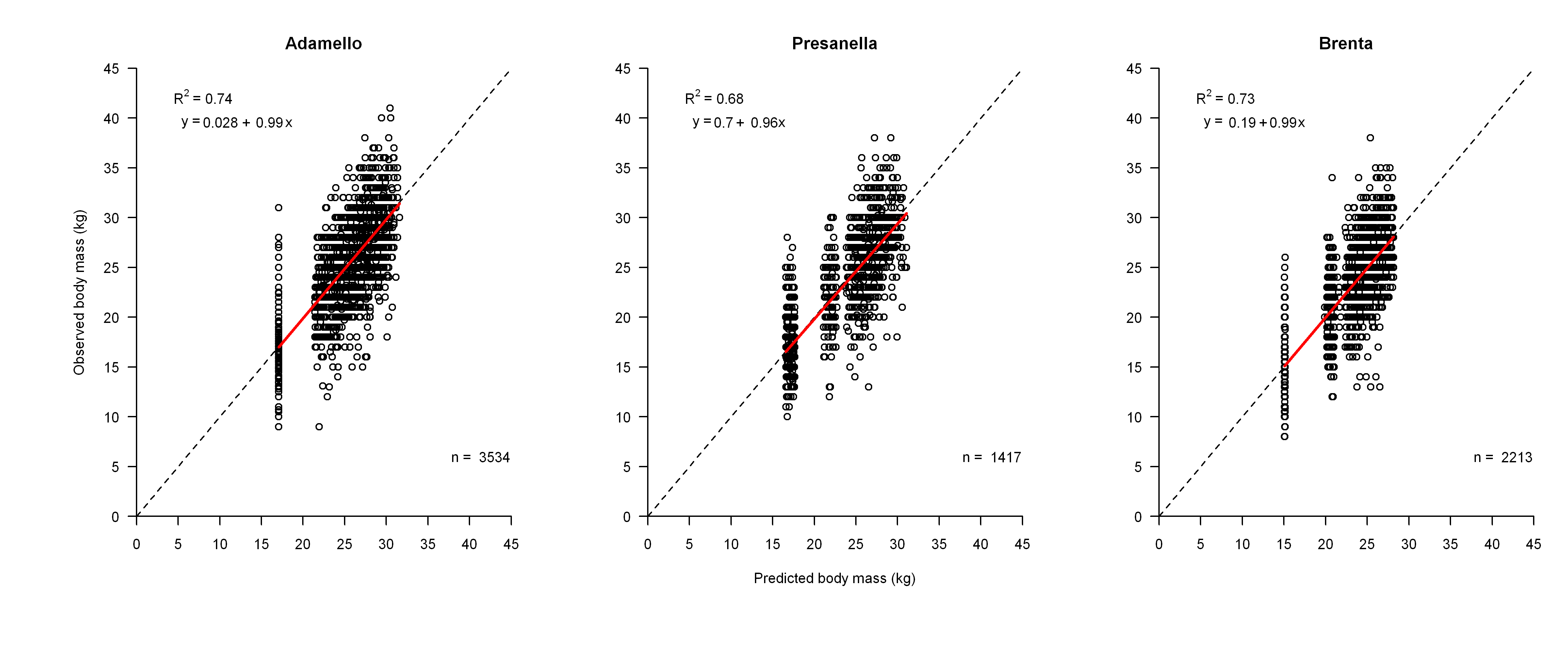

Supplement: Figure S4 — Plots of model fit for males. The relationship between observed body mass and predicted body mass by best model, in each site. Linear regressions plotted (red lines). Fitted line equations and R2 values shown. Dotted lines show 1∶1 relationship. (TIF) [file pone.0028002.s004.tif]

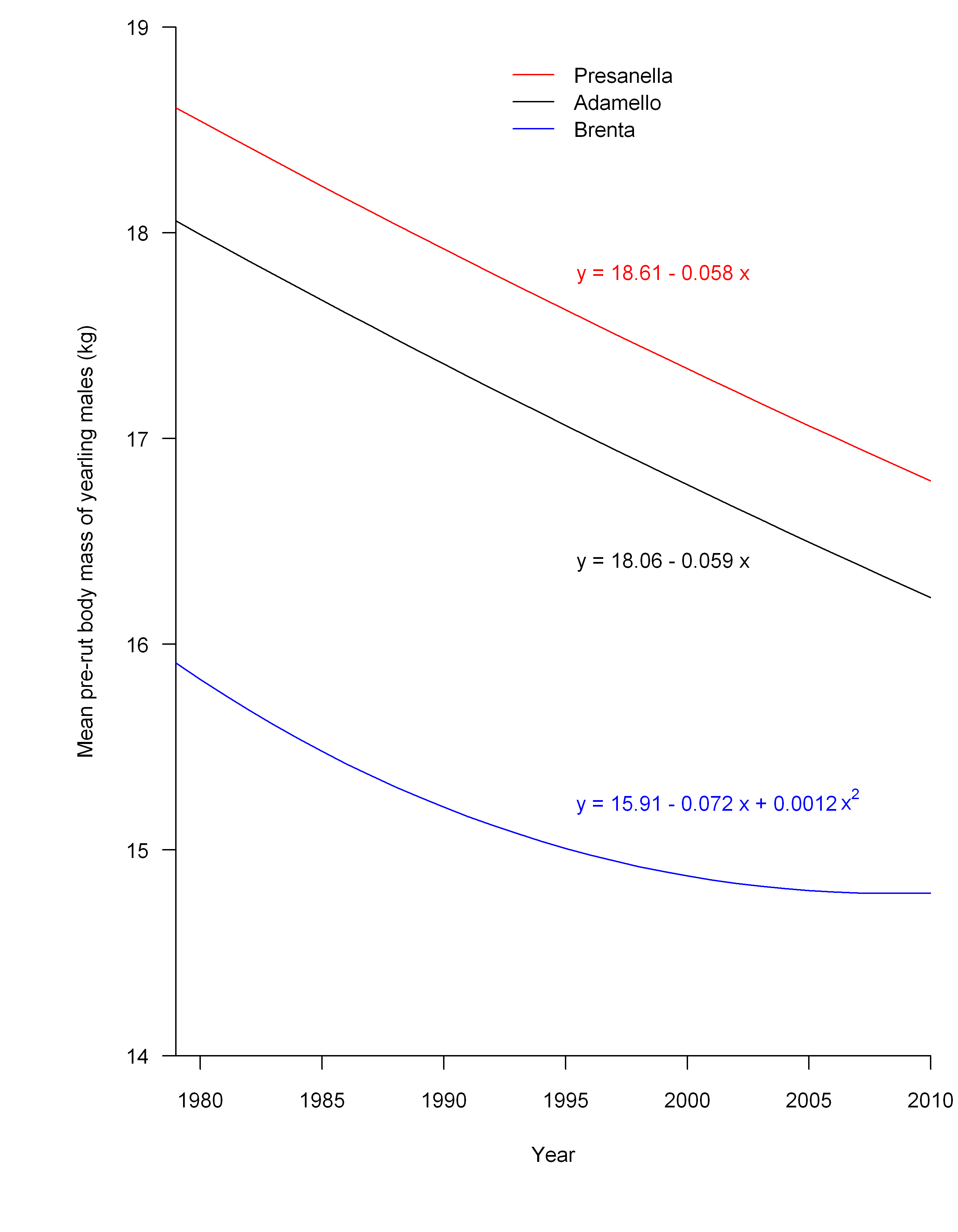

Supplement: Figure S5 — Change in predicted yearling male mass over time. Variation between predicted pre-rut body mass of yearling males (on day 300) with year in Presanella (red line), Adamello (black line) and Brenta (blue line). Line equations shown. There is a clear trend of decreasing body mass over the study period. (TIF) [file pone.0028002.s005.tif]
