# Supplementary material for: Contrasting Life Histories in Neighbouring Populations of a Large Mammal
Source: PLoS One. 2011 Nov 18;6(11):e28002. doi: 10.1371/journal.pone.0028002 (PMC3220718; doi:10.1371/journal.pone.0028002)
Supplement: Table S1 — Sex and age-specific chamois hunting quotas in Trento province. (DOC) [file pone.0028002.s006.doc]

**Table S1.** Sex and age-specific chamois hunting quotas in Trento province.

| Sex | Age class | Age (years) | Approximate proportion of quota (%) |
| --- | --- | --- | --- |
| Male | I | ≥6 | 16 |
| Male | II | 2 - 5 | 10 |
| Male | III | 1 | 22 |
| Female | I | ≥11 | 13 |
| Female | II | 2 - 10 | 17 |
| Female | III | 1 | 22 |
