# Supplementary material for: Contrasting Life Histories in Neighbouring Populations of a Large Mammal
Source: PLoS One. 2011 Nov 18;6(11):e28002. doi: 10.1371/journal.pone.0028002 (PMC3220718; doi:10.1371/journal.pone.0028002)
Supplement: Table S2 — Male and females data summary. (DOC) [file pone.0028002.s007.doc]

**Table S2.** Male and females data summary.

|  |  |  | **Males** | | | **Females** | | |
| --- | --- | --- | --- | --- | --- | --- | --- | --- |
| **Site** | **Year** | ***d*** | ***n*** | **Mean mass** | **SD of mass** | ***n*** | **Mean mass** | **SD of mass** |
| Adamello | 1979 | 0.011 | 96 | 24.4 | 5.7 | 70 | 20.4 | 4.5 |
| Adamello | 1980 | 0.011 | 63 | 23.8 | 5.8 | 51 | 21.8 | 4.4 |
| Adamello | 1981 | 0.01 | 59 | 24.1 | 5.7 | 65 | 20.7 | 3.9 |
| Adamello | 1982 | 0.01 | 51 | 23.6 | 4.9 | 54 | 20.1 | 3.2 |
| Adamello | 1983 | 0.011 | 68 | 22.6 | 4.7 | 62 | 20.9 | 3.6 |
| Adamello | 1984 | 0.011 | 81 | 23.2 | 4.5 | 70 | 20.7 | 4 |
| Adamello | 1985 | 0.01 | 91 | 22.5 | 4.4 | 66 | 21.1 | 4 |
| Adamello | 1986 | 0.01 | 76 | 22.7 | 5 | 65 | 20.2 | 3.2 |
| Adamello | 1987 | 0.011 | 92 | 22.9 | 5.1 | 71 | 20.3 | 3.2 |
| Adamello | 1988 | 0.012 | 89 | 24 | 4 | 76 | 20.9 | 3.7 |
| Adamello | 1989 | 0.012 | 77 | 22.4 | 4.8 | 94 | 20.9 | 3.3 |
| Adamello | 1990 | 0.013 | 105 | 22.8 | 5 | 87 | 20.3 | 4 |
| Adamello | 1991 | 0.015 | 114 | 23.6 | 5.4 | 111 | 21.5 | 3.1 |
| Adamello | 1992 | 0.015 | 111 | 22.5 | 5.6 | 134 | 20 | 3.4 |
| Adamello | 1993 | 0.018 | 123 | 21.4 | 5.8 | 114 | 19.5 | 3.9 |
| Adamello | 1994 | 0.02 | 110 | 22.1 | 5.7 | 118 | 18.5 | 3.2 |
| Adamello | 1995 | 0.023 | 165 | 22.1 | 5.5 | 131 | 19.8 | 3.8 |
| Adamello | 1996 | 0.023 | 163 | 23 | 5.1 | 134 | 20.2 | 3.4 |
| Adamello | 1997 | 0.021 | 144 | 22.4 | 5.3 | 121 | 20.1 | 3.4 |
| Adamello | 1998 | 0.021 | 123 | 22 | 5 | 98 | 18.8 | 3.2 |
| Adamello | 1999 | 0.021 | 156 | 22.4 | 5.6 | 126 | 19.5 | 3.4 |
| Adamello | 2000 | 0.022 | 147 | 21.7 | 5.9 | 151 | 18.9 | 4.4 |
| Adamello | 2001 | 0.02 | 132 | 23.6 | 6.2 | 130 | 19.4 | 4 |
| Adamello | 2002 | 0.02 | 136 | 21.3 | 5.1 | 115 | 19.3 | 4.1 |
| Adamello | 2003 | 0.02 | 130 | 21.9 | 5 | 101 | 18.9 | 4.1 |
| Adamello | 2004 | 0.019 | 106 | 23.2 | 5.3 | 77 | 21 | 4.4 |
| Adamello | 2005 | 0.015 | 107 | 23.5 | 5.8 | 69 | 19.6 | 4 |
| Adamello | 2006 | 0.016 | 129 | 23.1 | 5.7 | 95 | 19.5 | 4.1 |
| Adamello | 2007 | 0.017 | 130 | 21.6 | 5.3 | 94 | 18.5 | 4.1 |
| Adamello | 2008 | 0.015 | 93 | 23.5 | 6 | 75 | 20.2 | 3.7 |
| Adamello | 2009 | 0.017 | 139 | 23.3 | 5.3 | 75 | 19.3 | 3.9 |
| Adamello | 2010 | 0.016 | 133 | 22.6 | 5.9 | 90 | 19.7 | 4.1 |
|  |  | **Total** | 3539 |  | **Total** | 2990 |  |  |
| Presanella | 1979 | 0.008 | 21 | 23.7 | 6.5 | 23 | 21.6 | 4 |
| Presanella | 1980 | 0.009 | 25 | 25.1 | 5.7 | 27 | 21.6 | 4.1 |
| Presanella | 1981 | 0.013 | 34 | 23.4 | 4.4 | 32 | 21.5 | 3.8 |
| Presanella | 1982 | 0.014 | 37 | 23.6 | 5.3 | 29 | 21.1 | 4.1 |
| Presanella | 1983 | 0.011 | 37 | 22.8 | 5.4 | 25 | 21 | 3.1 |
| Presanella | 1984 | 0.009 | 21 | 23.5 | 6.7 | 23 | 21.3 | 3.5 |
| Presanella | 1985 | 0.012 | 28 | 21.4 | 5.8 | 28 | 20.4 | 4.3 |
| Presanella | 1986 | 0.01 | 15 | 24.6 | 5.3 | 32 | 21.7 | 4.4 |
| Presanella | 1987 | 0.012 | 44 | 21.4 | 4.9 | 40 | 21.2 | 3.6 |
| Presanella | 1988 | 0.014 | 41 | 21.9 | 4.5 | 42 | 20 | 3.5 |
| Presanella | 1989 | 0.014 | 47 | 21.8 | 5 | 41 | 20.5 | 3.7 |
| Presanella | 1990 | 0.013 | 28 | 24.1 | 5.6 | 44 | 20.2 | 3.6 |
| Presanella | 1991 | 0.013 | 49 | 22.3 | 5.4 | 29 | 20.7 | 3.4 |
| Presanella | 1992 | 0.015 | 44 | 23.1 | 6.6 | 41 | 20.1 | 3.8 |
| Presanella | 1993 | 0.016 | 49 | 21 | 5.4 | 29 | 20.3 | 3.3 |
| Presanella | 1994 | 0.017 | 50 | 21.8 | 5 | 51 | 20 | 3.4 |
| Presanella | 1995 | 0.017 | 49 | 22.6 | 5.4 | 48 | 19.6 | 3.8 |
| Presanella | 1996 | 0.018 | 50 | 22.4 | 4.7 | 45 | 19.3 | 3.1 |
| Presanella | 1997 | 0.02 | 55 | 23.9 | 5.2 | 50 | 19.9 | 3.3 |
| Presanella | 1998 | 0.019 | 44 | 22.4 | 5.6 | 57 | 20.4 | 4.1 |
| Presanella | 1999 | 0.017 | 52 | 20.5 | 5.7 | 60 | 19.3 | 3.8 |
| Presanella | 2000 | 0.02 | 58 | 21.6 | 6.3 | 47 | 19.9 | 4.1 |
| Presanella | 2001 | 0.02 | 62 | 23.6 | 5.2 | 58 | 20.9 | 4.3 |
| Presanella | 2002 | 0.018 | 55 | 22.3 | 6.1 | 52 | 18 | 3.9 |
| Presanella | 2003 | 0.02 | 57 | 21.4 | 5 | 60 | 19.2 | 3.8 |
| Presanella | 2004 | 0.018 | 57 | 22.6 | 5.6 | 49 | 21.3 | 4.1 |
| Presanella | 2005 | 0.015 | 42 | 23.1 | 6.1 | 38 | 18.2 | 4 |
| Presanella | 2006 | 0.016 | 50 | 22.6 | 6.3 | 55 | 19.1 | 3.6 |
| Presanella | 2007 | 0.017 | 50 | 23.2 | 5.8 | 42 | 19.7 | 3.3 |
| Presanella | 2008 | 0.015 | 42 | 20.5 | 5.4 | 40 | 18.5 | 3.4 |
| Presanella | 2009 | 0.019 | 65 | 24.1 | 4.2 | 41 | 19.7 | 3.4 |
| Presanella | 2010 | 0.019 | 68 | 22 | 5.2 | 51 | 19.6 | 3.4 |
|  |  | **Total** | 1426 |  | **Total** | 1329 |  |  |
| Brenta | 1979 | 0.012 | 69 | 23.2 | 6.1 | 54 | 20.3 | 4.5 |
| Brenta | 1980 | 0.012 | 62 | 21.9 | 6 | 53 | 19.7 | 3.8 |
| Brenta | 1981 | 0.014 | 65 | 21.9 | 4.3 | 46 | 19.4 | 3.3 |
| Brenta | 1982 | 0.013 | 74 | 20.5 | 4.6 | 41 | 18.7 | 4 |
| Brenta | 1983 | 0.013 | 42 | 18.7 | 5.2 | 43 | 18.2 | 3.3 |
| Brenta | 1984 | 0.013 | 70 | 20 | 4.5 | 50 | 17 | 3 |
| Brenta | 1985 | 0.013 | 75 | 20.2 | 5 | 59 | 16.8 | 3.3 |
| Brenta | 1986 | 0.012 | 44 | 20.7 | 4.9 | 66 | 17.5 | 2.7 |
| Brenta | 1987 | 0.013 | 61 | 19.6 | 4.5 | 62 | 17.8 | 3.4 |
| Brenta | 1988 | 0.015 | 50 | 20.1 | 4.8 | 62 | 18.8 | 3.3 |
| Brenta | 1989 | 0.015 | 71 | 21.7 | 6.1 | 66 | 17.6 | 3.7 |
| Brenta | 1990 | 0.016 | 81 | 21.6 | 5.2 | 70 | 17.3 | 3.8 |
| Brenta | 1991 | 0.016 | 84 | 21.9 | 6.2 | 75 | 17.9 | 4 |
| Brenta | 1992 | 0.014 | 79 | 21.1 | 5.7 | 76 | 17.9 | 3.8 |
| Brenta | 1993 | 0.015 | 66 | 21.7 | 5.5 | 83 | 17.3 | 3.8 |
| Brenta | 1994 | 0.017 | 54 | 20 | 6.8 | 81 | 16.9 | 4 |
| Brenta | 1995 | 0.017 | 65 | 22.6 | 5.5 | 71 | 17.2 | 4.1 |
| Brenta | 1996 | 0.019 | 64 | 20.2 | 6.5 | 88 | 17.7 | 4.1 |
| Brenta | 1997 | 0.019 | 80 | 21.7 | 5.1 | 67 | 18.3 | 3.7 |
| Brenta | 1998 | 0.019 | 85 | 20.1 | 5.5 | 86 | 18 | 3.4 |
| Brenta | 1999 | 0.017 | 75 | 19.9 | 4.8 | 75 | 17.6 | 3.4 |
| Brenta | 2000 | 0.017 | 78 | 21.1 | 5.6 | 83 | 17.3 | 4.1 |
| Brenta | 2001 | 0.018 | 86 | 20.4 | 5.7 | 88 | 17.8 | 3.7 |
| Brenta | 2002 | 0.019 | 61 | 20.5 | 5.5 | 76 | 18.1 | 3.9 |
| Brenta | 2003 | 0.018 | 85 | 20.4 | 5.1 | 83 | 17 | 3.9 |
| Brenta | 2004 | 0.018 | 69 | 21.2 | 5.5 | 66 | 17.7 | 3.7 |
| Brenta | 2005 | 0.019 | 64 | 21 | 5.5 | 65 | 18.1 | 3.9 |
| Brenta | 2006 | 0.018 | 79 | 19.9 | 5.1 | 68 | 17 | 3.5 |
| Brenta | 2007 | 0.018 | 77 | 21 | 5.1 | 76 | 18.3 | 3.7 |
| Brenta | 2008 | 0.015 | 80 | 21.6 | 5.3 | 34 | 17 | 3.4 |
| Brenta | 2009 | 0.017 | 73 | 22.4 | 5.4 | 43 | 17.5 | 4.1 |
| Brenta | 2010 | 0.014 | 69 | 20 | 6.1 | 40 | 16.2 | 3.8 |
|  |  | **Total** | 2237 |  | **Total** | 2096 |  |  |

Population density estimates*, d* (individuals m^-2^), for different sites and years calculated by dividing the number of individuals culled by the area of suitable habitat. Sample sizes (*n*), mean body mass (kg) and standard deviation of body mass shown for different sites and years.
