# Supplementary material for: Contrasting Life Histories in Neighbouring Populations of a Large Mammal
Source: PLoS One. 2011 Nov 18;6(11):e28002. doi: 10.1371/journal.pone.0028002 (PMC3220718; doi:10.1371/journal.pone.0028002)
Supplement: Table S3 — Model selection results from cubic spline model fitted to male chamois body mass data. (DOC) [file pone.0028002.s008.doc]

**Table S3. Model selection results from cubic spline model fitted to male chamois body mass data.**

| Model | K | LL | AIC | ΔAIC |
| --- | --- | --- | --- | --- |
| **M(*α*_0_(y^2^),*β*_0_(y^2^),S)** | **51** | **-16998.1** | **34098.2** | **0.0** |
| **M(*α*_0_(y),*β*_0_(y^2^),S)** | **48** | **-17002.8** | **34101.5** | **3.4** |
| **M(*α*_0_(y^2^),*β*_0_(y),S)** | **48** | **-17003.3** | **34102.7** | **4.5** |
| M(*α*_0_(y),*β*_0_(y),S) | 45 | -17008.6 | 34107.2 | 9.0 |
| M(*α*_0_(y^2^),*β*_0_(d),S) | 48 | -17010.3 | 34116.7 | 18.5 |
| M(*α*_0_(y),*β*_0_(d),S) | 45 | -17014.2 | 34118.3 | 20.1 |
| M(*α*_0_(y^2^),*β*_0_,S) | 45 | -17019.1 | 34128.2 | 30.1 |
| M(*α*_0_(d),*β*_0_(d),S) | 45 | -17022.3 | 34134.5 | 36.3 |
| M(*α*_0_(y),*β*_0_,S) | 42 | -17024.2 | 34132.5 | 34.3 |
| M(*α*_0_(d),*β*_0_(y^2^),S) | 48 | -17025.6 | 34147.1 | 48.9 |
| M(*α*_0_(d),*β*_0_(y),S) | 45 | -17031.8 | 34153.6 | 55.4 |
| M(*α*_0_(d),*β*_0_,S) | 42 | -17041.6 | 34167.2 | 69.0 |
| M(*α*_0_,*β*_0_(y^2^),S) | 45 | -17060.1 | 34210.3 | 112.1 |
| M(*α*_0_,*β*_0_(y),S) | 42 | -17081.1 | 34246.3 | 148.1 |
| M(*α*_0_,*β*_0_(d),S) | 42 | -17086.5 | 34257.0 | 158.8 |
| M(*α*_0_,*β*_0_,S) | 39 | -17124.3 | 34326.7 | 228.5 |
| M(*α*_0_(y),*β*_0_(y)) | 15 | -18889 | 37808.5 | 3710.3 |
| M(*α*_0_(y^2^),*β*_0_(y)) | 16 | -18889 | 37810.1 | 3711.9 |
| M(*α*_0_(y),*β*_0_(y^2^)) | 16 | -18889 | 37810.3 | 3712.1 |
| M(*α*_0_(y^2^),*β*_0_(y^2^)) | 17 | -18889 | 37812.1 | 3713.9 |
| M(*α*_0_(y),*β*_0_) | 14 | -18893 | 37814.6 | 3716.4 |
| M(*α*_0_(y),*β*_0_(d)) | 15 | -18893 | 37815.5 | 3717.3 |
| M(*α*_0_(y^2^),*β*_0_) | 15 | -18893 | 37816.6 | 3718.4 |
| M(*α*_0_(y^2^),*β*_0_(d)) | 16 | -18892 | 37816.7 | 3718.5 |
| M(*α*_0_(d),*β*_0_(y)) | 15 | -18918 | 37865.2 | 3767.0 |
| M(*α*_0_(d),*β*_0_(y^2^)) | 16 | -18917 | 37866.3 | 3768.1 |
| M(*α*_0_(d),*β*_0_(d)) | 15 | -18921 | 37871.6 | 3773.4 |
| M(*α*_0_,*β*_0_(y^2^)) | 15 | -18937 | 37904.5 | 3806.3 |
| M(*α*_0_,*β*_0_(y)) | 14 | -18939 | 37905.2 | 3807.0 |
| M(*α*_0_(d),*β*_0_) | 14 | -18941 | 37910 | 3811.9 |
| M(*α*_0_,*β*_0_(d)) | 14 | -18973 | 37973.3 | 3875.1 |
| M(*α*_0_,*β*_0_) | 13 | -18975 | 37976.3 | 3878.1 |

Models are distinguished by the functional forms of *α*_0_ and *β*_0_. Specifically, we allowed *α*_0_ and *β*_0_ to be constant across years (*α*_0_; *β*_0_), vary linearly with year (*α*_0_(y); *β*_0_(y)), quadratically with year (*α*_0_(y^2^); *β*_0_(y^2^)) or linearly with population density (*α*_0_(d); *β*_0_(d)). We either treated sites separately (denoted by S) or ignored site-effects. Maximum log-likelihoods (LL) and ΔAICs are shown for each site. Maximum log-likelihoods (LL) and ΔAICs are shown for each site. The most parsimonious models for each site are highlighted in bold (i.e. have a ΔAIC value that is ≤6 and lower than all simpler nested versions; see Richards [40]). K is the number of parameters in each model.
