# Supplementary material for: Contrasting Life Histories in Neighbouring Populations of a Large Mammal
Source: PLoS One. 2011 Nov 18;6(11):e28002. doi: 10.1371/journal.pone.0028002 (PMC3220718; doi:10.1371/journal.pone.0028002)
Supplement: Table S4 — Model selection results from cubic spline model fitted to female chamois body mass data. (DOC) [file pone.0028002.s009.doc]

**Table S4.** Model selection results from cubic spline model fitted to female chamois body mass data.

| Model | K | LL | AIC | ΔAIC |
| --- | --- | --- | --- | --- |
| **M(*α*_0_(y^2^),*β*_0_(y),S)** | **48** | **-15186.8** | **30469.6** | **0.0** |
| M(*α*_0_(y^2^),*β*_0_(y^2^),S) | 51 | -15186.4 | 30474.9 | 5.3 |
| M(*α*_0_(y),*β*_0_(y^2^),S) | 48 | -15194.0 | 30484.0 | 14.4 |
| M(*α*_0_(y),*β*_0_(y),S) | 45 | -15197.5 | 30485.0 | 15.4 |
| M(*α*_0_(d),*β*_0_(y^2^),S) | 48 | -15195.8 | 30487.6 | 18.1 |
| M(*α*_0_(y^2^),*β*_0_(d),S) | 48 | -15197.6 | 30491.2 | 21.6 |
| M(*α*_0_(y),*β*_0_(d),S) | 45 | -15202.1 | 30494.2 | 24.7 |
| M(*α*_0_(y^2^),*β*_0_,S) | 45 | -15205.2 | 30500.4 | 30.9 |
| M(*α*_0_(y),*β*_0_,S) | 42 | -15211.1 | 30506.2 | 36.7 |
| M(*α*_0_,*β*_0_(y^2^),S) | 45 | -15210.2 | 30510.5 | 40.9 |
| M(*α*_0_(d),*β*_0_(y),S) | 45 | -15216.0 | 30522.0 | 52.4 |
| M(*α*_0_(d),*β*_0_(d),S) | 45 | -15226.2 | 30542.5 | 72.9 |
| M(*α*_0_,*β*_0_(y),S) | 42 | -15229.3 | 30542.5 | 72.9 |
| M(*α*_0_(d),*β*_0_,S) | 42 | -15236.3 | 30556.5 | 86.9 |
| M(*α*_0_,*β*_0_(d),S) | 42 | -15260.9 | 30605.7 | 136.1 |
| M(*α*_0_,*β*_0_,S) | 39 | -15282.4 | 30642.8 | 173.3 |
| M(*α*_0_(y^2^),*β*_0_(y)) | 16 | -17151.9 | 34335.9 | 3866.3 |
| M(*α*_0_(y^2^),*β*_0_(y^2^)) | 17 | -17152.0 | 34338.0 | 3868.4 |
| M(*α*_0_(y^2^),*β*_0_(d)) | 16 | -17153.6 | 34339.3 | 3869.7 |
| M(*α*_0_(y),*β*_0_(y^2^)) | 16 | -17154.6 | 34341.3 | 3871.7 |
| M(*α*_0_,*β*_0_(y^2^)) | 15 | -17161.1 | 34352.2 | 3882.6 |
| M(*α*_0_(y),*β*_0_(d)) | 15 | -17161.3 | 34352.6 | 3883.0 |
| M(*α*_0_(y^2^),*β*_0_) | 15 | -17161.7 | 34353.4 | 3883.8 |
| M(*α*_0_(y),*β*_0_) | 14 | -17169.2 | 34366.5 | 3896.9 |
| M(*α*_0_(d),*β*_0_) | 14 | -17172.8 | 34373.6 | 3904.0 |
| M(*α*_0_,*β*_0_(y)) | 14 | -17172.8 | 34373.6 | 3904.0 |
| M(*α*_0_(d),*β*_0_(d)) | 15 | -17172.7 | 34375.4 | 3905.9 |
| M(*α*_0_(d),*β*_0_(y)) | 15 | -17174.8 | 34379.5 | 3909.9 |
| M(*α*_0_(d),*β*_0_(y^2^)) | 16 | -17174.7 | 34381.3 | 3911.7 |
| M(*α*_0_(y),*β*_0_(y)) | 15 | -17188.0 | 34406.0 | 3936.4 |
| M(*α*_0_,*β*_0_(d)) | 14 | -17189.2 | 34406.4 | 3936.8 |
| M(*α*_0_,*β*_0_) | 13 | -17218.1 | 34462.1 | 3992.5 |

Models are distinguished by the functional forms of *α*_0_ and *β*_0_. Specifically, we allowed *α*_0_ and *β*_0_ to be constant across years (*α*_0_; *β*_0_), vary linearly with year (*α*_0_(y); *β*_0_(y)), quadratically with year (*α*_0_(y^2^); *β*_0_(y^2^)) or linearly with population density (*α*_0_(d); *β*_0_(d)). We either treated sites separately (denoted by S) or ignored site-effects. Maximum log-likelihoods (LL) and ΔAICs are shown for each site. Maximum log-likelihoods (LL) and ΔAICs are shown for each site. The most parsimonious models for each site are highlighted in bold (i.e. have a ΔAIC value that is ≤6 and lower than all simpler nested versions; see Richards [40]). K is the number of parameters in each model.
